# Hepatitis B infection (HBsAg and HBeAg) status among women attending antenatal care at public healthcare facilities of South Africa, 2017

**DOI:** 10.1371/journal.pgph.0003567

**Published:** 2025-01-22

**Authors:** Shelina Moonsamy, Pavitra Pillay, Beverley A. Singh, Adrian Puren, John W. Ward, Nishi Prabdial-Sing

**Affiliations:** 1 Centre for Vaccines and Immunology, National Institute for Communicable Diseases, a division of the National Health Laboratory Service, Johannesburg, South Africa; 2 Department of Biomedical and Clinical Technology, Faculty of Health Sciences, Durban University of Technology, Durban, South Africa; 3 Centre for HIV and STIs, National Institute for Communicable Diseases, a division of the National Health Laboratory Service, Johannesburg, South Africa; 4 Coalition for Global Hepatitis Elimination, Task Force for Global Health, Decatur, Georgia, United States of America; 5 Hubert Department of Global Health, Rollins School of Public Health, Emory University, Atlanta, Georgia, United States of America; 6 Department of Medical Virology, Faculty of Health Sciences, School of Pathology, University of the Witwatersrand, Johannesburg, South Africa; University of Cape Town, SOUTH AFRICA

## Abstract

Eight years after WHO adopted a resolution to eliminate hepatitis B by the year 2030, the disease remains a global public health concern, with vertical transmission of HBV being a major obstacle to this goal. Our study aimed to determine the HBV infection status of pregnant women in South Africa at a national level to evaluate the risk of vertical transmission and provide evidence for public health decision-making. We conducted HBsAg testing on 1,942 HIV-uninfected and 2,312 HIV-infected pregnant women from South Africa’s public health sector in 2017, followed by HBeAg testing on HBsAg-positive samples. Our data were stratified by five-year age groups and province. The overall HBV prevalence was 11.24% (478/4,254), significantly higher among HIV-infected women (15.83%) compared to HIV-uninfected women (5.77%, p = 0.007). HBV prevalence was highest among women 40–44 years (14.00%) and in Limpopo Province (19.35%). Coinfection rates of HIV-HBV ranged from 14.00% to 17.00% among women 15–44 years, and provincially, rates were highest in Limpopo, North West, and Western Cape Provinces (>20%). HBeAg prevalence among HBsAg positive women was 9.48%, with higher rates among HIV infected women (11.29%) compared to HIV-uninfected women (7.34%, p = 0.7931). HBeAg prevalence was highest among women 15–19 (10.81%), 20–24 (11.88%) and 40–44 years (25.00%), and provincially, highest in North West (26.67%). Our findings highlight the significant prevalence of HBV infection among pregnant women in 2017, emphasising concerns about vertical transmission, particularly given the high prevalence of HBeAg among HBsAg-positive women. We advocate for the prompt implementation of a universal birth dose of the HBV vaccine in South Africa to augment existing vaccination schedules and mitigate the risk of vertical transmission, thereby advancing progress towards WHO elimination targets.

## 1. Introduction

Eight years after WHO adopted a resolution to eliminate hepatitis B by the year 2030, the disease remains a global public health concern [1]. A major obstacle to this goal is vertical transmission of the hepatitis B virus (HBV) from mother to child, a route that contributes substantially to the burden of chronic HBV infections. Nearly 90% of vertically infected infants develop chronic infection, which carries a one-in-four risk of premature death from liver failure or hepatocellular carcinoma [2–5]. As part of its elimination strategy, WHO has set a target to reduce the prevalence of hepatitis B surface antigen (HBsAg) to less than 0.1% among children under five years of age by 2030, a target aligned with the United Nations Sustainable Development Goals [6]. HBsAg remains the key diagnostic marker for active HBV infection and a cornerstone for measuring progress toward elimination [7,8].

HBV infections in children arise from both vertical transmission at birth and horizontal transmission during early childhood. South Africa, despite its long-standing implementation of an infant HBV vaccination program since 1995, continues to face challenges in eliminating HBV transmission [9,10]. Our previous study identified an HBsAg positivity rate of 4.83% among children under five years old during the period 2015–2019, underscoring ongoing gaps in prevention strategies [11].

Vertical transmission risk is heightened in HBsAg-positive pregnant women who are also positive for hepatitis B e-antigen (HBeAg), a marker of increased viral replication and transmissibility [12–15]. While HBV vaccination programs have been instrumental in reducing HBV infections, universal hepatitis B birth dose (HepB-BD) has not been implemented in South Africa [4,16]. Current strategies rely on administering HepB-BD and hepatitis B immunoglobulin (HBIG) to infants born to HBsAg-positive mothers, alongside maternal antiviral therapy during pregnancy [17].

HIV coinfection further complicates the burden of HBV in South Africa. Shared transmission routes likely contribute to the high rates of HBV-HIV coinfection, with approximately 8%–10% of individuals living with HIV globally also infected with HBV [18,19]. Coinfected individuals are at higher risk of severe liver complications, including accelerated progression to cirrhosis and hepatocellular carcinoma [18,20–22]. Certain interventions targeted at minimising the burden of HIV, such as the use of tenofovir as an antiviral treatment option, have also demonstrated efficacy against HBV, highlighting the potential dual benefits of these strategies in managing both infections [22–25].

To address the ongoing risk of vertical transmission, it is critical to assess the prevalence of active HBV infection among pregnant women. Understanding the national burden of maternal HBV infection is essential to inform interventions, including universal HepB-BD vaccination. This study aimed to determine the HBV infection status of pregnant women in South Africa at a national level to evaluate the risk of vertical transmission and provide evidence for public health decision-making.

## 2. Methods

### 2.1. Ethics and approvals

Our study utilised anonymised retrospective samples, each assigned a unique identifier. The authors had no access to any information that could identify individual participants throughout the study period. At the time of sample collection, participants provided consent for their samples to be archived for possible future studies.

We obtained ethics approval from the Faculty of Health Sciences, Institutional Research Ethics Committee (IREC 069/20) of the Durban University of Technology, Durban, South Africa. Access to the archived samples and data was approved via the Academic Affairs and Research Management System (PR20254) of the National Health Laboratory Service (NHLS), Johannesburg, South Africa, and via The Centre for Disease Control and Prevention (Accession #: CGH-SOAFR-7/19/22-86d1f), Atlanta, United States of America.

### 2.2 Study design and population

This study adopted a retrospective cross-sectional design. The samples were from participants of the 2017 antenatal HIV sentinel survey [26,27]. Briefly, since 1990, antenatal HIV surveys have been systematically conducted to facilitate HIV surveillance among pregnant women attending antenatal care at public healthcare facilities in South Africa [27]. The primary objective of these surveys has been to estimate HIV prevalence trends over time among pregnant women aged 15 to 49 years receiving antenatal care. Initially conducted annually from 1990 to 2015, the survey transitioned to a biennial schedule from 2015 onwards [28]. Post-testing, antenatal samples were first archived at the various testing laboratories and were subsequently archived at the National Institute for Occupational Health in Johannesburg, South Africa, from where they were retrieved for HBV testing. [26].

### 2.3 Sample size

We calculated our sample size based on the reported formula for a prevalence study [29]. To apply this formula, we needed the most recent estimates of HBV prevalence among HIV-uninfected and HIV-infected pregnant women. In order to obtain these estimates, we used our reported HBV prevalence (HBsAg test positivity rate of 2019) among women of child-bearing age as a proxy for prevalence amongst South African pregnant women, and the reported prevalence among HIV-infected and HIV-uninfected pregnant women of Western Cape Province in 2008 [30,31]. We used these prevalence rates to estimate the national prevalence of HBV among HIV-infected and HIV-uninfected pregnant women and subsequently calculated the appropriate sample sizes for these groups of women. On obtaining the final sample sizes, we subsequently calculated the representative proportions applicable to the 2017 antenatal survey by HIV status. The HIV antenatal survey data were obtained and accessed on October 26, 2021. We applied the calculated proportions in Stata/SE 18.0 (Texas, USA) to randomly select samples by HIV status and province of South Africa to ensure the attainment of a nationally representative sample. Considering the anticipated possibility of some samples being insufficient or unavailable for various reasons, we selected an additional 5% of the calculated sample sizes.

### 2.4 Testing methodology

The Abbott Architect i1000SR (Abbott Diagnostics, Illinois, USA) was used as the testing platform to perform HBsAg and HBeAg tests on HBsAg positive samples. The assays for each of the serological markers were performed according to the manufacturer’s instructions.

### 2.5 Data analyses

Data obtained following HBV testing (S1 Data) were used for analyses in Stata/SE 18.0 (Texas, USA).

We first analysed the HBsAg marker to determine the overall HBsAg test positivity rate (HBV prevalence), and prevalence by five-year age groups and by province. This was followed by analysis to assess whether there were significant differences in HBV prevalence between HIV-uninfected and HIV-infected individuals across five-year age groups and provinces. Given that our study was performed on samples from 2017, we conducted additional analysis to determine the prevalence of HBsAg among women who would remain within childbearing ages by 2024 (15–42 years).

With regards to HBeAg, we observed during testing that some HBsAg positive samples were insufficient for HBeAg testing. Consequently, it was necessary to ascertain the number of such samples to calculate the overall HBeAg test positivity rate and determine if these differed significantly between HIV-uninfected and HIV-infected individuals, overall and by five-year age groups and province.

For all statistical inferences, we applied the two-sample test of proportions with a significance level (α) of 0.05 [32].

## 3. Results

### 3.1 Sample size

In 2019, HBsAg test positivity rates were reported as 2.11%, 3.73%, 6.13%, 6.85%, 7.19%, 7.20% and 6.69% among females aged 15–19, 20–24, 25–29, 30–34, 35–39, 40–44 and 45–49 years old respectively [31]. The average HBsAg test positivity rate was therefore calculated to be 5.70%. Based on the reported HBsAg prevalence in 2008, of 3.10% overall, 2.80% among HIV-uninfected pregnant women and 3.40% among HIV-infected pregnant women, we estimated HBsAg prevalence to be 5.10% and 6.30% among HIV-uninfected and HIV-infected pregnant women respectively [30].

The sample size for HIV-uninfected pregnant women was subsequently calculated to be ≈1,860 (1.96^2^ * 0.051 (1 − 0.051)/0.01^2^), and the sample size for HIV-infected pregnant women was calculated to be ≈2,268 (1.96^2^ * 0.063 (1 − 0.063)/0.01^2^). The 2017 HIV antenatal dataset consisted of 32,716 samples, with 22,358 HIV negative results and 10,358 positive results [26,27]. Our sample size for HIV-uninfected individuals was therefore ≈8.32% of 22,358 (proportion of 0.0832), and for HIV-infected individuals was ≈21.90% of 10,358 (proportion of 0.219). Applying these proportions, we subsequently obtained the sample numbers required for HBV testing per province and HIV status. Through adding the additional 5%, we obtained sample sizes of 1953 and 2381 for HIV-uninfected and HIV-infected individuals, respectively. Table 1 shows the sample numbers obtained from our sample size calculations and the sample numbers tested per province and HIV status (Table 1a), and the sample numbers tested per five-year age groups (Table 1b).

**Table 1:** Calculated and tested sample size by province (a), and tested sample size by five-year age groups (b).

(a)							(b)			
Province	Calculated sample size by province^a^			Tested sample size by province^b^			Age group	Tested sample size by age group^b^		
	HIV-uninfected	HIV-infected	Total	HIV-uninfected	HIV-infected	Total		HIV-uninfected	HIV-infected	Total
**Eastern Cape**	223	298	521	223	300	523	15–19	330	113	443
**Free State**	153	196	349	153	197	350	20–24	573	462	1,035
**Gauteng**	273	341	614	294	342	636	25–29	449	658	1,107
**KwaZulu-Natal**	404	742	1,146	403	746	1,149	30–34	279	546	825
**Limpopo**	169	136	305	172	138	310	35–39	120	308	428
**Mpumalanga**	150	234	384	177	243	420	40–44	29	71	100
**North West**	136	137	273	136	136	272	45–49	1	3	4
**Northern Cape**	103	59	162	103	84	187	Unknown	161	151	312
**Western Cape**	250	124	374	281	126	407				
**Total**	**1,861**	**2,267**	**4,128**	**1,942**	**2,312**	**4,254**	**Total**	**1,942**	**2,312**	**4,254**

^a^The calculated sample sizes excluding the additional 5% to account for insufficiency or unavailability.

^b^The tested sample sizes including the additional 5% to account for insufficiency or unavailability.

Overall, inclusive of the additional 5% of samples selected, we tested 126 samples more than what was required, 81 HIV negative and 45 HIV positive samples. At the provincial level, mirroring the HIV antenatal survey, the bulk of the cases were from KwaZulu-Natal (1,149, 27.01%) (Table 1a). Among different age groups, the majority of women fell within the 20 to 34-year-old bracket (2,967, 69.75%), while there were only four cases in the 45 to 49-year-old category.

### 3.2 HBsAg prevalence

**Fig 1:**
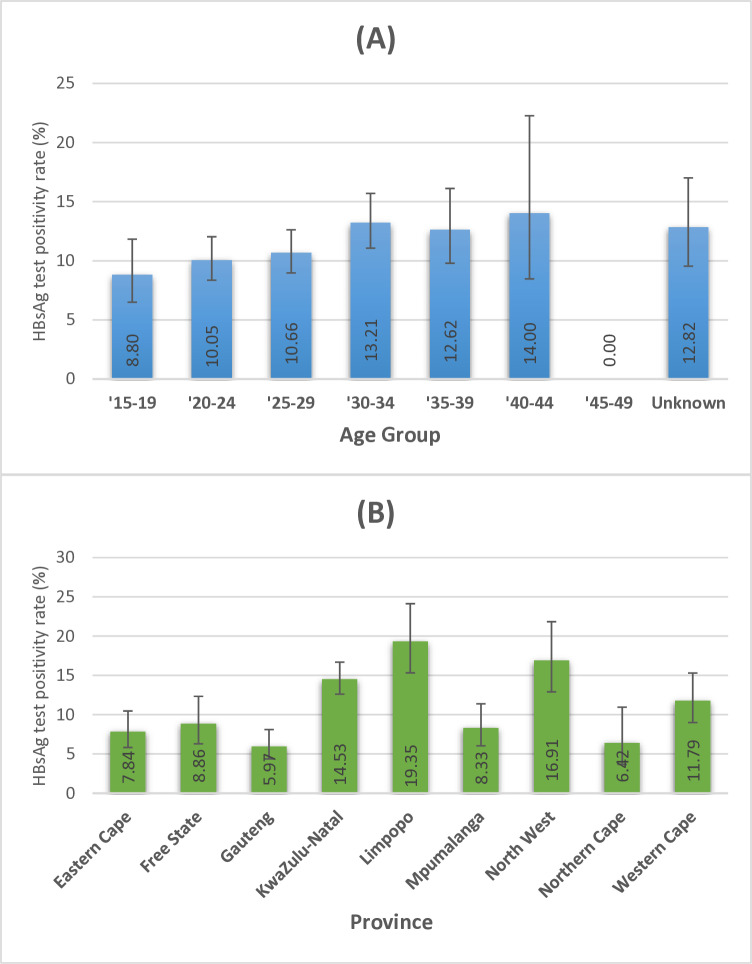
HBsAg test positivity rate (%) and 95% confidence intervals among pregnant women in 2017 by five-year age groups (a), and province (b).

From the total of 4,254 samples subjected to HBsAg testing, 478 yielded positive results, indicating an overall HBV prevalence (HBsAg test positivity rate) of 11.24% (95% CI: 10.30%–12.22%). The distribution of cases across five-year age groups and by province is illustrated in Fig 1.

Excluding women aged 45–49 years due to their limited representation, HBV prevalence rates ranged from 8.80% among women 15–19 years old to 14.00% among women 40–44 years old (Fig 1a). At the provincial level, HBV prevalence varied considerably, ranging from 5.97% in Gauteng to 19.35% in Limpopo (>3-fold increase compared to Gauteng).

The HBsAg results, as well as the proportion of positive HBsAg results (HBsAg positivity) by HIV status within five-year age groups and across the different provinces is shown in Table 2.

**Table 2:** The frequency of HBsAg results and HBsAg positivity by HIV status within five-year age groups (a) and across the different provinces (b).

(a)									
Age group	HIV-uninfected (#)			HIV-infected (#)			Proportion HBsAg positive among HIV-uninfected individuals (95% CI)	Proportion HBsAg positive among HIV-infected individuals (95% CI)	P-value*
	HBsAg results			HBsAg results					
	Negative (#)	Positive (#)	Total (#)	Negative (#)	Positive (#)	Total (#)			
**’15–19**	309	21	330	95	18	113	0.06 (0.04–0.10)	0.16 (0.10–0.24)	0.3122
**’20–24**	541	32	573	390	72	462	0.06 (0.04–0.08)	0.16 (0.13–0.19)	0.1606
**’25–29**	425	24	449	564	94	658	0.05 (0.04–0.080)	0.14 (0.12–0.17)	0.2287
**’30–34**	261	18	279	455	91	546	0.06 (0.04–0.100)	0.17 (0.14–0.20)	0.2347
**’35–39**	116	4	120	258	50	308	0.03 (0.01–0.09)	0.16 (0.13–0.21)	0.484
**’40–44**	27	2	29	59	12	71	0.07 (0.02–0.24)	0.17 (0.10–0.27)	0.7188
**’45–49**	1	0	1	3	0	3	0.00 (–)	0.00 (–)	**
**Unknown**	150	11	161	122	29	151	0.07 (0.04–0.120)	0.19 (0.14–0.26)	0.3516
**Total**	1830	112	1942	1946	366	2312	0.06 (0.05–0.07)	0.16 (0.14–0.17)	**0.007**
**(b)**									
**Province**	**HIV-uninfected (#)**			**HIV-infected (#)**			**Proportion HBsAg positive among HIV-uninfected individuals (95% CI)**	**Proportion HBsAg positive among HIV-infected individuals (95% CI)**	P-value*
	**HBsAg results**			**HBsAg results**					
	**Negative (#)**	**Positive (#)**	**Total (#)**	**Negative (#)**	**Positive (#)**	**Total (#)**			
**Eastern Cape**	215	8	223	267	33	300	0.04 (0.02–0.07)	0.11 (0.08–0.15)	0.5472
**Free State**	146	7	153	173	24	197	0.05 (0.02–0.09)	0.12 (0.08–0.18)	0.5938
**Gauteng**	284	10	294	314	28	342	0.03 (0.02–0.06)	0.08 (0.06–0.12)	0.5868
**KwaZulu-Natal**	376	27	403	606	140	746	0.07 (0.05–0.10)	0.19 (0.16–0.22)	0.1291
**Limpopo**	149	23	172	101	37	138	0.13 (0.09–0.19)	0.27 (0.20 0.35)	0.2004
**Mpumalanga**	163	14	177	222	21	243	0.08 (0.05–0.13)	0.09 (0.06–0.13)	0.9177
**North West**	124	12	136	102	34	136	0.09 (0.05–0.15)	0.25 (0.18–0.33)	0.2406
**Northern Cape**	100	3	103	75	9	84	0.03 (0.01–0.09)	0.11 (0.06–0.19)	0.675
**Western Cape**	273	8	281	86	40	126	0.03 (0.01–0.06)	0.32 (0.24–0.40)	0.0923
**Total**	1830	112	1942	1946	366	2312	0.06 (0.05–0.07)	0.16 (0.14–0.17)	**0.007**

*P-value generated from the test of proportions between HIV-uninfected HBsAg positive individuals versus HIV-infected HBsAg positive individuals, alpha = 0.05. Bold indicates significant difference.

**Insufficient observations to generate a P-value.

Overall, HBsAg positivity was significantly higher among HIV-infected women (15.83%) compared to HIV-uninfected women (5.77%, P = 0.007) (Table 2). Comparing HBsAg positivity between HIV-uninfected and HIV-infected women by age groups and provinces, although consistently higher among HIV-infected women, the differences were not statistically significant (Table 2). Regarding HIV-HBV coinfection, prevalence was high among women 15 to 44 years old (14%–17%). By province, HIV-HBV coinfection rates were highest in Limpopo, North West, and Western Cape Provinces (above 20%) compared to other provinces (Table 2).

On further analysis to determine HBsAg prevalence among women 15–42 years old (a group considered within child-bearing ages in 2024), we observed a prevalence of 11.11% (Table 3).

**Table 3:** The frequency and percentage of HBsAg results among women considered within child-bearing ages in 2024.

Age group	HBsAg negative # (%)	HBsAg positive # (%)	Total
15–42	3,487 (88.89%)	436 (11.11%)	3,923
43–49	17 (89.47%)	2 (10.53%)	19
Unknown	272 (87.18%)	40 (12.82%)	312
**Total**	**3,776 (88.76%)**	**478 (11.24%)**	**4,254**

### 3.3 HBeAg prevalence among HBsAg positive pregnant women

Out of the 478 samples that tested positive for HBsAg, 464 (97.07%) samples were tested for HBeAg as 14 were insufficient for further testing. Of 464 HBsAg positive samples, 44 (9.48%, 95% CI: 6.97% to 15.52%) were positive for HBeAg. Although HBeAg positivity was higher among HIV-infected women, the difference was not statistically significant (P = 0.7931, Table 4).

**Table 4:** HBeAg results on HBsAg positive samples stratified by HIV status.

HIV results	HBeAg results			Proportion HBeAg positive (95% CI)	P-value*
	Negative	Positive	Total		
**HIV-uninfected**	101	8	109	0.07 (0.04–0.14)	0.7931
**HIV-infected**	319	36	355	0.10 (0.07–0.14)	
**Total**	420	44	464	0.09 (0.07–0.13)	–

*P-value generated from the test of proportions between HIV-uninfected HBsAg positive individuals versus HIV-infected HBsAg positive individuals, alpha = 0.05.

Stratifying HBeAg results by five-year age groups and province, we observed the highest test positivity rates among ages 15–24 years and 40–44 years, and in Eastern Cape, Mpumalanga and North West Provinces (Table 5).

**Table 5:** HBeAg results and proportion positive by five-year age groups (a) and province (b).

		HBeAg			
		Negative (#)	Positive (#)	Total (#)	Proportion HBeAg positive (95% CI)
**Age group (a)**	**15–19**	33	4	37	0.11 (0.04–0.26)
	**20–24**	89	12	101	0.12 (0.07–0.20)
	**25–29**	108	5	113	0.04 (0.02–0.100
	**30–34**	99	9	108	0.08 (0.04–0.15)
	**35–39**	50	4	54	0.07 (0.03–0.18)
	**40–44**	9	3	12	0.25 (0.08–0.55)
	**Unknown**	32	7	39	0.18 (0.09–0.33)
**Province (b)**	**Eastern Cape**	34	6	40	0.15 (0.07–0.30)
	**Free State**	29	1	30	0.03 **(**0.00–0.20)
	**Gauteng**	28	2	30	0.07 (0.02–0.23)
	**KwaZulu-Natal**	154	11	165	0.07 (0.04–0.120
	**Limpopo**	55	5	60	0.08 **(**0.04–0.19)
	**Mpumalanga**	29	6	35	0.17 (0.08–0.33)
	**North West**	33	12	45	0.27 (0.16–0.41)
	**Northern Cape**	12	0	12	0.00 (–)
	**Western Cape**	46	1	47	0.02 (0.00–0.14)
	**Total**	420	44	464	0.09 (0.07–0.13)

Where the number of observations were sufficient for statistical analyses, no significant differences were observed in HBeAg positivity between HIV-uninfected and HIV-infected pregnant women (S1 Table).

## 4. Summary and discussion

We conducted HBV testing on 13.00% of samples collected during the 2017 HIV antenatal sentinel survey, specifically from women attending public healthcare facilities for antenatal care in that year. Among the samples tested, HBV prevalence was notably high at 11.24%. The overall high HBV prevalence observed among these pregnant women raises significant and ongoing concerns regarding vertical transmission of HBV, prompting urgent need to explore and implement either national or targeted interventions to mitigate associated risks. This concern is further corroborated by the notable HBeAg positivity among women who tested positive for HBsAg, indicating an increased risk of transmitting HBV to their babies.

Comparing HBV prevalence among HIV-uninfected versus HIV-infected pregnant women, we observed significantly higher rates among the latter group of women. The increased prevalence in HIV-infected women was anticipated due to the common transmission routes shared by HIV and HBV infections and the higher risk of active HBV infection for persons with compromised immunity from HIV infection [18]. Beyond shared transmission routes, other factors may have contributed to this finding, including HIV-related immunosuppression altering the natural course of HBV infection and delayed HBV clearance in co-infected individuals [33–36]. Nevertheless, the rates were unexpectedly almost three times higher, raising significant concern since individuals with HIV-HBV coinfections have a much higher risk of progressive liver disease [18]. Considering the shared transmission routes, the implementation of supplementary strategies to mitigate HIV infection may simultaneously aid in alleviating the burden of HBV [23]. Reflecting upon the heightened focus HIV has already received and the implementation of interventions aimed at reducing its transmission, it is conceivable that these efforts may have inadvertently influenced the prevalence of HBV infection, implying that we might have observed a higher prevalence of HBV among pregnant women than what was observed in this study [24]. In 2010, South Africa introduced the use of tenofovir as an intervention for the prevention of mother-to-child transmission of HIV, a drug that has also been reported as safe and effective in reducing vertical transmission of HBV [37–43]. Therefore, women who were co-infected with HBV and HIV and were treated with tenofovir for the prevention of vertical transmission of HIV might also have benefited from antiviral medication to prevent vertical transmission of HBV. However, tenofovir alone cannot prevent vertical transmission of HBV and the need for timely HBV-BD is strongly recommended, together with HBIG, if and where available [44,45]. It is important to highlight here that this applies specifically to women who were co-infected with HBV and HIV and treated with tenofovir following diagnosis of HIV. Consequently, among women who are infected with HBV but not HIV, the risk of vertical transmission of HBV remains unchanged, as there are no specific measures in place to reduce this risk.

Our findings on HBsAg and HBeAg prevalence across the different age groups demonstrated the highest rates among women aged 40–44 years. However, after excluding women 45–49 years old due to limited representation, prevalence rates were still considered high (>10%) for HBsAg among women aged 20 years and older, as well as for HBeAg among women aged 15–24 years. It is important to note that women aged 23 years and older were born prior to the introduction of the HBV vaccine into South Africa’s EPI schedule in 1995, during a period when hepatitis B was highly endemic in the country [9]. Despite a slightly lower HBsAg prevalence among women aged 15–19 years, a group eligible for vaccination at birth, the rate is still concerning as it indicates acquired HBV infection in this group. One of the reasons may be linked to suboptimal vaccine coverage rates reported for South Africa, which may have contributed to some of these women not having received any HBV vaccinations or not completing three-dose course and subsequently becoming infected [11,31]. However, it must be emphasised that even if these women received vaccinations as infants, the possibility of having contracted HBV through vertical infection at birth cannot be ruled out since the first HBV vaccine dose has traditionally been administered at six weeks of age in South Africa [37]. Among those co-infected with HIV, all women 15–44 years showed high co-infection prevalence, indicative of the shared risks of transmission inclusive of multiple sexual partners, as well as gender-based violence that drive HIV transmission in the country [46–49].

Despite our data representing a cohort of antenatal women from seven years ago, HBsAg prevalence remained high among women who would still be considered child-bearing in 2024. This is extremely worrying as it indicates a similar risk of vertical transmission of HBV currently compared to seven years ago.

Analysing HBsAg prevalence at a provincial level, we observed notably high rates (>10%) in KwaZulu-Natal, Limpopo, North West, and Western Cape Provinces. Among the latter three provinces, the highest rates of HIV-HBV co-infection (>20%) were also observed. Our findings, specifically for Limpopo Province with the highest HBV prevalence of 19.35%, compared well with our reported HBsAg test positivity rate of 19.52% in the same province during the same year [31]. Analysing HBeAg prevalence, the highest rates (15%) were seen in Eastern Cape, Mpumalanga and North West Provinces. Examining both HBsAg and HBeAg prevalence, North West was identified as the province with a notably high HBsAg prevalence and the highest HBeAg prevalence. On the other hand, while we observed a high HBsAg prevalence in Western Cape Province, we also observed the lowest HBeAg prevalence in this province. We have previously highlighted the need for thorough investigations to potentially identify the underlying reasons for provincial differences; however, this remains a task that still needs to be addressed [11,31]. Furthermore, given the significantly higher HBsAg prevalence among HIV-infected individuals overall, integrating HIV and HBV services in the public health sector of South Africa may aid in finding common risk factors across provinces.

We acknowledge that the retrospective cross-sectional design of our study relies on previously collected data and archived samples, limiting control over the quality and completeness of data. Variability in sample storage and handling conditions could have affected the accuracy of test results. The study relied on a dataset originally collected for HIV surveillance, which may lack variables critical to HBV-specific analyses, such as vaccination history or prior HBV exposure. Our observations specifically pertain to pregnant women attending antenatal visits at public healthcare facilities in 2017. As a result, the findings may not be generalisable to pregnant women accessing private healthcare or those not attending antenatal care. Sample size calculations were based on proxy estimates of HBV prevalence, including data from 2019 on women of child-bearing age and 2008 data from the Western Cape Province. These may not accurately reflect the national HBV prevalence among pregnant women in 2017, potentially affecting the precision of prevalence estimates. Our study focused on HBsAg and HBeAg, and other markers like HBV DNA and anti-HBc were not included. This might underestimate the true burden of HBV. Our study did not factor in CD4 counts among HIV-infected women, which may have provided some insights into the interplay between HIV-related immunosuppression and HBV infection. Despite these limitations, this study marks the first nationwide HBV testing initiative targeting pregnant women in South Africa, addressing a gap in existing research and providing valuable insights into HBV prevalence among pregnant women in the country and subsequently, the associated vertical transmission risks.

## 5. Conclusion

Although the data is representing a cohort of antenatal women from seven years ago, our findings demonstrated a significant proportion of HBV infection among pregnant women of 2017 in South Africa. This speaks to the suboptimal vaccine coverage for the past thirty years. Consequently, we draw the conclusion that the risk of vertical transmission of HBV poses a substantial threat to achieving the WHO elimination targets. To address this concern, we advocate for the prompt implementation of a universal birth dose of the HBV vaccine in South Africa in addition to the standard doses administered at 6, 10, and 14 weeks of age. Furthermore, testing of more recent antenatal samples (from the HIV antenatal surveys) can further determine whether we are closing the immunity gap with improved vaccine coverage, enhanced awareness regarding HBV transmission routes and the potential consequences of acquiring HBV infection at early ages. Such measures may contribute significantly to reducing the overall burden of HBV disease, not only in South Africa but also in other countries facing a high HBV disease burden.

## Supporting information

S1 Data HBV data on samples randomly selected from the 2017 HIV antenatal survey, stratified by province and age group. (ZIP)

S1 Table HBeAg positivity among HIV-uninfected and HIV-infected HBsAg positive individuals. (DOCX)
