# Supplementary material for: Hepatitis B infection (HBsAg and HBeAg) status among women attending antenatal care at public healthcare facilities of South Africa, 2017
Source: PLOS Glob Public Health. 2025 Jan 22;5(1):e0003567. doi: 10.1371/journal.pgph.0003567 (PMC11753652; doi:10.1371/journal.pgph.0003567)
Supplement: S1 Table — (DOCX) [file pgph.0003567.s002.docx]

| **Age Group and Province Distribution** | | **HIV Results** | | | | **P Value*** |
| --- | --- | --- | --- | --- | --- | --- |
|  |  | **HIV-uninfected** | | **HIV-infected** | |  |
|  |  | **HBeAg Results** | | **HBeAg Results** | |  |
|  |  | **Nonreactive # (%)** | **Reactive # (%)** | **Nonreactive # (%)** | **Reactive # (%)** |  |
| **Age Group (a)** | **15-19** | 19 (90.48) | 2 (9.52) | 14 (87.5) | 2 (12.5) | 0.9251 |
|  | **20-24** | 28 (90.32) | 3 (9.68) | 61 (87.14) | 9 (12.86) | 0.8908 |
|  | **25-29** | 22 (100) | 0 (-) | 86 (94.51) | 5 (5.49) | ** |
|  | **30-34** | 16 (88.89) | 2 (11.11) | 83 (92.22) | 7 (7.78) | 0.8942 |
|  | **35-39** | 4 (100) | 0 (-) | 46 (92) | 4 (8) | ** |
|  | **40-44** | 2 (100) | 0 (-) | 7 (70) | 3 (30) | ** |
|  | **Unknown** | 10 (90.91) | 1 (9.09) | 22 (78.57) | 6 (21.43) | ** |
| **Province (b)** | **Eastern Cape** | 7 (87.5) | 1 (12.5) | 27 (84.38) | 5 (15.62) | ** |
|  | **Free State** | 6 (100) | 0 (-) | 23 (95.83) | 1 (4.17) | ** |
|  | **Gauteng** | 8 (100) | 0 (-) | 20 (90.91) | 2 (9.09) | ** |
|  | **KwaZulu-Natal** | 24 (88.89) | 3 (11.11) | 130 (94.2) | 8 (5.8) | 0.7773 |
|  | **Limpopo** | 20 (86.96) | 3 (13.04) | 35 (94.59) | 2 (5.41) | 0.7682 |
|  | **Mpumalanga** | 14 (100) | 0 (-) | 15 (71.43) | 6 (28.57) | ** |
|  | **North West** | 11 (91.67) | 1 (8.33) | 22 (66.67) | 11 (33.33) | ** |
|  | **Northern Cape** | 3 (100) | 0 (-) | 9 (100) | 0 (-) | ** |
|  | **Western Cape** | 8 (100) | 0 (-) | 38 (97.440) | 1 (2.56) | ** |
| **Total** | | **101 (92.66)** | **8 (7.34)** | **319 (89.86)** | **36 (10.14)** | **0.7931** |
| **P-Value generated from the test of proportions between HIV-uninfected HBeAg positive individuals versus HIV-infected HBeAg positive individuals, alpha = 0.05 **Insufficient observations to generate a P-Value* | | | | | | |
|  |  |  |  |  |  |  |
